# Repeat aortic valve replacement (AVR) for pseudoaneurysm of mitral-aortic intervalvular fibrosa (P-MAIVF) repair due to hemolytic anemia 6 years after AVR: a case report

**DOI:** 10.1186/s44215-023-00036-3

**Published:** 2023-04-19

**Authors:** Masanobu Yamauchi, Kazuma Kanetsuki, Tomoki Hanada, Satoshi Kamihira

**Affiliations:** Department of Cardiovascular Surgery, Shimane Prefectural Central Hospital, 4 chome 1-1, Himebara, Izumo City, Shimane 693-8555 Japan

**Keywords:** Pseudoaneurysm, Mitral-aortic intervalvular fibrosa, Infective endocarditis, Aortic valve replacement

## Abstract

**Background:**

Pseudoaneurysm of the mitral-aortic intervalvular fibrosa (P-MAIVF) after infective endocarditis and/or valve replacement is rarely reported, and transesophageal echocardiography and cardiac multidetector computed tomography are useful for diagnosis. Surgery is mostly recommended to prevent fatal complications.

**Case presentation:**

A 61-year-old man underwent repeat aortic valve replacement (AVR) with repair of a P-MAIVF due to hemolytic anemia 6 years after AVR, and 4 months after treatment of sepsis and an infected abdominal aortic aneurysm. Two years after the surgery, the patient is alive and well with no recurrence.

**Conclusions:**

The present case was considered to be a very rare case in which surgery was performed because the blood flow entering and leaving the P-MAIVF contacted the prosthetic valve ring, resulting in hemolysis, severe anemia, and heart failure.

**Supplementary Information:**

The online version contains supplementary material available at 10.1186/s44215-023-00036-3.

## Background

Pseudoaneurysm of mitral-aortic intervalvular fibrosa (P-MAIVF) is relatively rare and is more likely to occur after infective endocarditis (IE) and/or aortic valve replacement (AVR) [1–5]. Surgery is mostly recommended to prevent fatal complications [1, 3–5]. We report a case of repeat AVR with repair of a P-MAIVF due to hemolytic anemia 6 years after AVR, and 4 months after treatment of sepsis and an infected abdominal aortic aneurysm (iAAA).

## Case presentation

Fifty-five-year-old man underwent AVR (St. Jude Medical valve, SJM 25 mm, Abbott Laboratories, Little Canada, MN) for congenital aortic bicuspid valve and severe aortic stenosis, followed by outpatient follow-up. Six years after AVR, he was admitted to our hospital with sepsis due to methicillin-sensitive *Staphylococcus aureus* and an iAAA (33 mm in size). Blood tests showed white blood cell count (WBC) 6340/μL (neutrophils 89%) and C-reactive protein (CRP) 28.9 mg/dL. Transthoracic echocardiography (TTE) during hospitalization showed no periprosthetic leakage and prosthetic valve endocarditis (PVE) findings. Contrast-enhanced computed tomography (CT) showed no lesions suspected of being a P-MAVIF or an abscess on the dorsal aortic root and no change in the iAAA. After blood cultures were confirmed to be negative three times, the CRP level was measured as 0.5 mg/dL, and the brain natriuretic hormone (BNP) level was measured as 12 pg/mL, and the patient was discharged on the 25th day of hospitalization under oral antibiotic medication (sulbactam/ampicillin, SBT/ABPC) following intravenous antibiotic therapy (cefazoline, CEZ). Four months later, he was admitted to our hospital for shortness of breath and jaundice due to hemolytic anemia, and was referred to our department for further evaluation. A pseudoaneurysm was found behind the aortic valve on transesophageal echocardiography (TEE), and hemolysis due to the prosthetic valve was suspected.

**Fig. 1:**
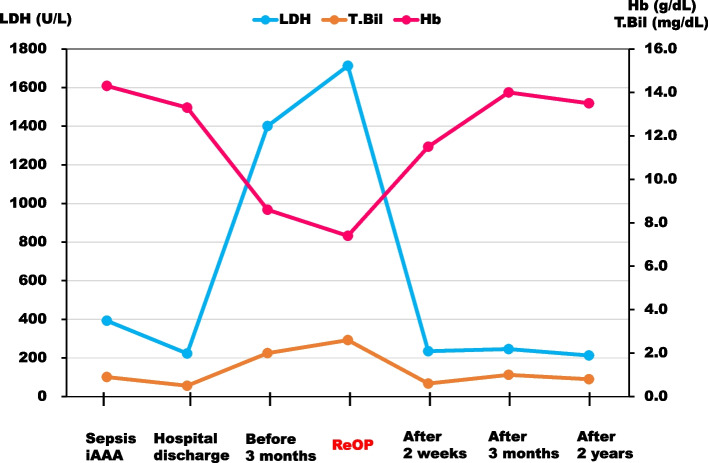
Changes in laboratory data. LDH: lactate dehydrogenase, T.Bil: total-bilirubin, Hb: hemoglobin

**Fig. 2:**
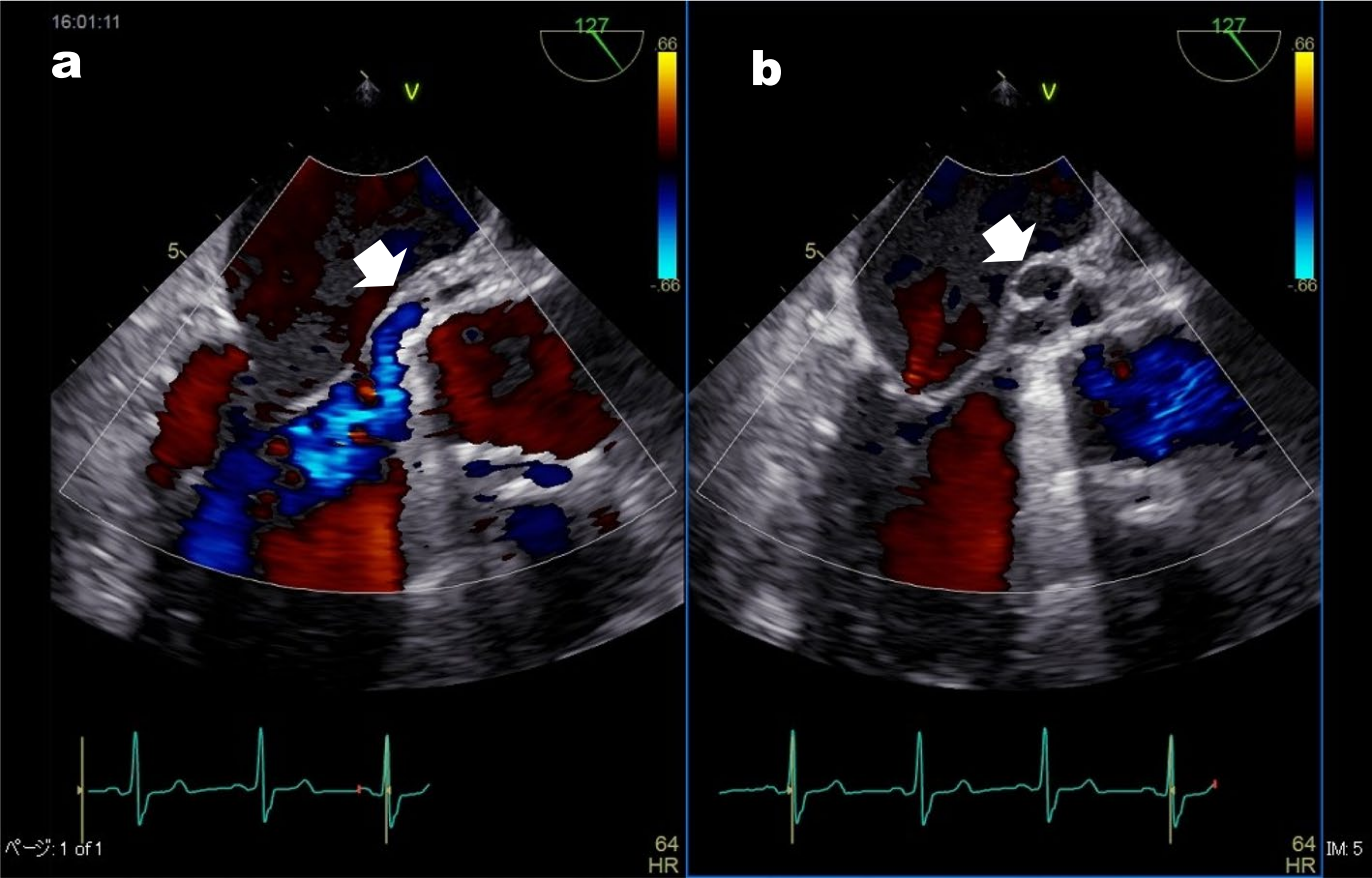
Transesophageal echocardiography (TEE). TEE showed a 13.4-mm × 23.6 mm × 12.1 mm pseudoaneurysm with a septum posterior to the aortic valve and blood flow communicating with the pseudoaneurysm and left ventricle, a characteristic finding of P-MAIVF with systolic expansion (**b**, arrow) and diastolic collapse (**a**, arrow). There was blood flow entering the P-MAIVF during systole and leaving from the P-MAIVF to the LVOT during diastole

**Fig. 3:**
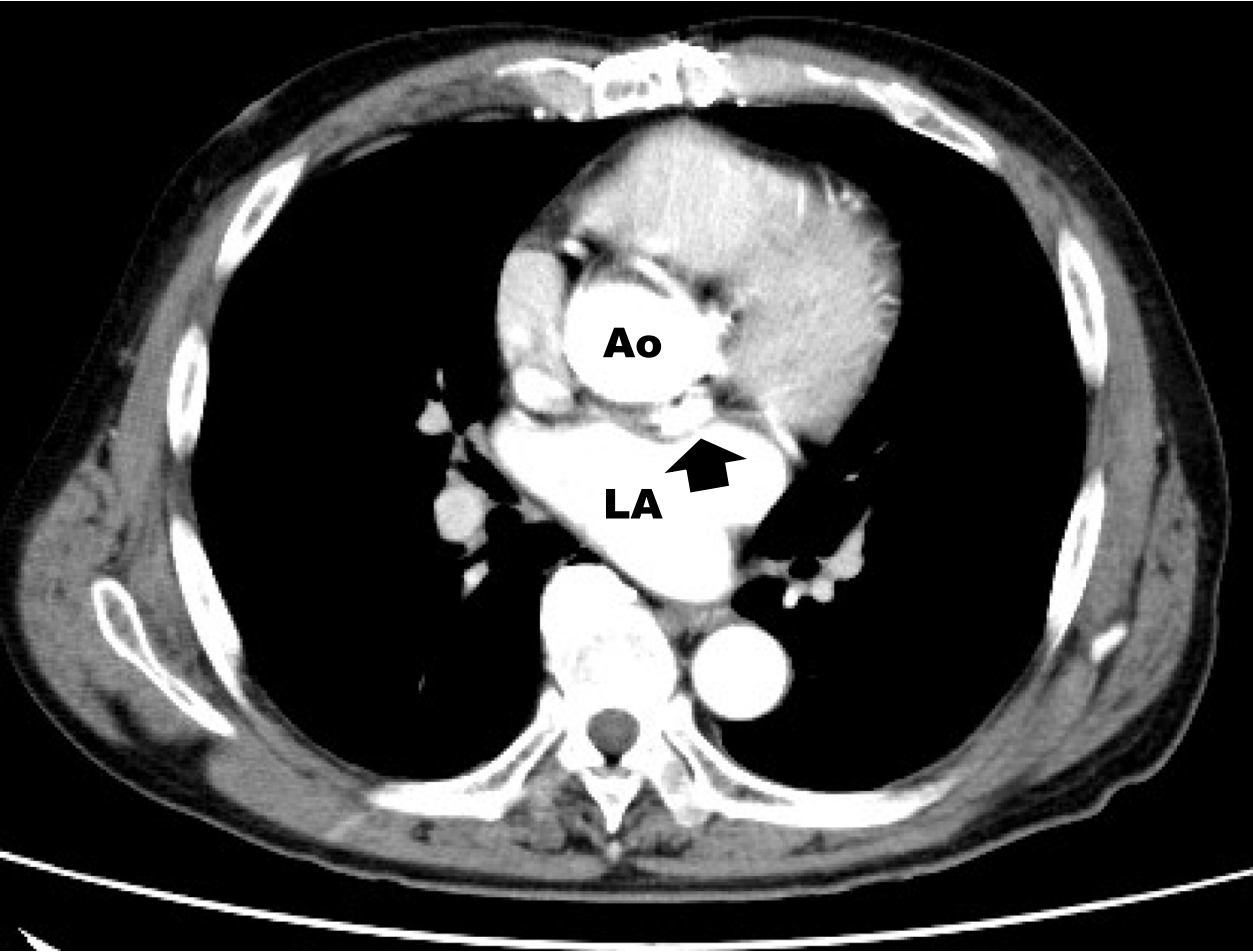
Multidetector computed tomography (MDCT). MDCT showed a pseudoaneurysm (24x10 mm in size, with internal septum) at the dorsal aortic root of the left coronary cusp and noncoronary cusp valve ring (arrow)

On admission, the vital signs were stable, and there was no heart murmur or mild jaundice. Blood tests showed hemoglobin 7.4 g/dL, total-bilirubin 2.6 mg/dlL, aspartate aminotransferase 75 U/L, lactate dehydrogenase (LDH) 1713 U/L (LDH isozyme 1 44.1%, LDH isozyme 2 39.8%), haptoglobin < 10 mg/dL, WBC 3570/μL (neutrophils 69%), CRP 0.04 mg/dL, and BNP 85 pg/mL (Fig. 1). TTE showed mild aortic regurgitation from the dorsal circumference of the prosthetic valve (which was actually diastolic blood flow from a pseudoaneurysm to the left ventricular outflow tract [LVOT]) with normal LV function, and the transmitral valve blood flow waveform showed a pseudonormal pattern. TEE showed no periprosthetic leakage and a 13.4 mm × 23.6 mm × 12.1 mm pseudoaneurysm with a septum posterior to the aortic valve and blood flow communicating with the pseudoaneurysm and left ventricle (LV), a characteristic finding of P-MAIVF with systolic expansion (Fig 2b; Additional file 1, TEE video) and diastolic collapse (Fig.2a). Multidetector computed tomography (MDCT) showed a pseudoaneurysm (24 × 10 mm in size, with internal septum) at the dorsal aortic root of the left coronary cusp and noncoronary cusp (NCC) valve ring, as in TEE (Fig.3).

**Fig. 4:**
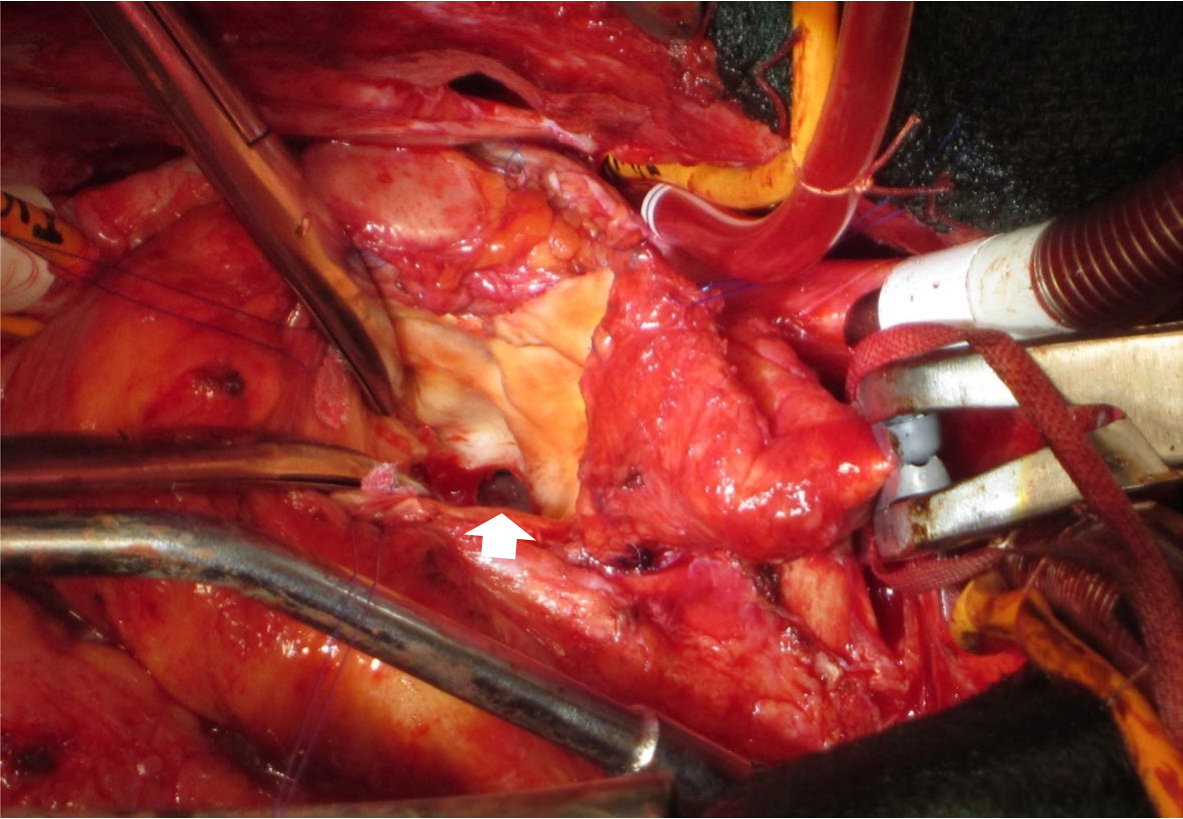
Surgical photograph. A 20 × 15 mm defect was found just below the noncoronary cusp valve ring (arrow)

He underwent repeat AVR with P-MAIVF repair. The sternum was reopened, and cardiopulmonary bypass was established. The operative findings showed no dehiscence between the prosthetic valve ring and the native aortic valve ring, and after removing the prosthesis, a 20 mm × 15 mm defect was confirmed for the first time in the LVOT just below the NCC of the native aortic valve ring (Fig.4). In addition, there were no macroscopic findings of active infection around the defect, and the bacterial culture results in the P-MAIVF cavity during surgery were negative. The defect was closed with a 28 × 23 mm bovine pericardial patch, and a biological prosthesis (Inspiris 23 mm, Carpenteir-Edwards pericardial valve, Edwards Lifesciences Corporation, Irvine, CA) was implanted in the supra-annular position. The aortic clamp time was 157 min, and the cardiopulmonary bypass time was 235 min. The postoperative course was good and the patient was discharged 12 days after surgery. Two years after the surgery, blood tests showed that hemoglobin was 13.5 g/dL, total-bilirubin was 0.8 mg/dL, LDH was 213 U/L and BNP was 28 pg/mL (Fig. 1) and the patient is alive and well with no recurrence.

## Discussion

The MAIVF is a fibrous skeleton between the aortic and mitral valves that lacks blood flow and is prone to forming pseudoaneurysms in patients after IE and/or AVR and with aortic bicuspid valves [1–4]. Regarding symptoms, patients can be asymptomatic (9%), or show signs of infection (39%), dyspnea and heart failure (16%), chest pain (10%), or cerebral embolism or systemic embolism (12%) [1]. If a pseudoaneurysm ruptures to the left atrium (LA) or aorta, severe heart failure may occur; if it ruptures in the pericardial sac, cardiac tamponade may occur; if it compresses a coronary artery, angina pectoris or myocardial infarction may occur; and a thrombus of pseudoaneurysm may cause a cerebral embolism or systemic embolism [1, 3]. The present case was considered to be a rare case in which surgery was performed because the blood flow entering and leaving the P-MAIVF contacted the prosthetic valve ring, resulting in hemolysis, severe anemia, and heart failure. *Staphylococcus aureus* and *Streptococcus* spp*.* are the most common organisms that cause IE [1, 3]. Sudhakar et al. reported that 35 patients (76%) had a history of endocarditis at some point, among 46 patients with prosthetic valves [1]. The diagnosis is first made by TTE, which has a low sensitivity of 43%, while the sensitivity of TEE is as high as 90% [5]. In addition, P-MAIVF has the characteristic findings of systolic expansion (mean area ± standard deviation 4.1 ± 3.4 cm^2^) and diastolic collapse (1.8 ± 2.2 cm^2^) [5]. Compared with P-MAIVFs, aortic ring abscesses are significantly smaller and do not exhibit pulsatility, as mentioned above [3, 5]. Color Doppler flow imaging shows no flow through the abscess [3, 5]. Furthermore, MDCT facilitates understanding of the anatomical relationship between the P-MAIVF and the surrounding LVOT, LA, and aorta.

Although many reports have recommended surgical or catheter closure as early as possible after diagnosis [1, 5], recent reports recommend observation in asymptomatic patients or those without IE [2, 3]. Grimaldi et al. reported that asymptomatic patients without previous known IE or valve regurgitation who were conservatively treated showed a good clinical outcome. Surgery is recommended in cases of active IE, pseudoaneurysm larger than 3 cm, congenital aortic bicuspid valve, aortic regurgitation, presence of fistula to the LA or aorta, thrombus in P-MAIVF or compression of coronary or pulmonary arteries [1]. The most common surgical methods are AVR and P-MAIVF repair [1–4]. Recently, catheter-based closure using coils, plugs, and transcatheter AVR (TAVR) has also been reported [4]. In surgical high-risk groups or when conservative treatment is chosen due to lack of patient consent, careful follow-up with TEE is recommended [1–3, 5].

## Supplementary Information

**Additional file 1:** Video of TEE showing no periprosthetic leakage and a 13.4 mm x 23.6 mm x 12.1 mm pseudoaneurysm with a septum posterior to the aortic valve and blood flow communicating with the pseudoaneurysm and left ventricle, a characteristic finding of P-MAIVF with systolic expansion and diastolic collapse.

## Data Availability

All data analyzed during this study are included in this published article.

## Abbreviations

AVR: Aortic valve replacement
P-MAIVF: Pseudoaneurysm of mitral-aortic intervalvular fibrosa
IE: Infective endocarditis
iAAA: Infected abdominal aortic aneurysm
WBC: White blood cell count
CRP: C-reactive protein
PVE: Prosthetic valve endocarditis
CT: Computed tomography
BNP: Brain natriuretic peptide
SBT/ABPC: Sulbactam/ampicillin
CEZ: Cefazoline
TEE: Transesophageal echocardiography
LDH: Lactate dehydrogenase
TTE: Transthoracic echocardiography
LVOT: Left ventricular outflow tract
LV: Left ventricle
MDCT: Multidetector computed tomography
NCC: Noncoronary cusp
LA: Left atrium
TAVR: Transcatheter aortic valve replacement.
